# The relationship between plasma serglycin levels and the diagnosis of diabetic retinopathy

**DOI:** 10.1002/jcla.23663

**Published:** 2020-12-12

**Authors:** Layi Wang, Yin Han, Xiajun Wang

**Affiliations:** ^1^ Ningbo Eye Hospital Ningbo China

**Keywords:** biomarker, diabetic retinopathy, diagnosis, SRGN

## Abstract

**Background:**

Diabetic retinopathy (DR), a microvascular complication which is closely related to diabetes, remains the leading cause of vision loss around the world among older adults. Serglycin (SRGN) was known as a hematopoietic cell granule proteoglycan, exerting its function in the formation of mast cell secretory granules and mediates the storage of various compounds in secretory vesicles. The present study illustrates the potential clinical value and experimental mechanisms of SRGN in the DR.

**Methods:**

Firstly, the mRNA expression and protein expression of SRGN in plasma samples from NPDR, PDR patients, type 2 diabetes mellitus (T2Dm) cases, and healthy controls were measured by qPCR and Western blotting assays, respectively. Then, the potentials of SRGN functioning as a diagnostic indicator in DR were verified by the receiver operating characteristic (ROC) analysis. We established in vitro DR model of human retinal endothelial cells through high‐glucose treatment. The expression of SRGN and its mechanisms of regulating cellular processes were illustrated subsequently.

**Results:**

Our data revealed that SRGN was dramatically upregulated in NPDR and PDR cases compared with healthy controls and T2DM patients; meanwhile, the expression of SRGN was further increased in the PDR group with regard to the NPDR group. Furthermore, the ROC analysis demonstrated that SRGN could distinguish the DR cases from type 2 diabetes mellitus (T2DM) patients and healthy controls with the area under the curve (AUC) of 0.7680 (95% CI = 0.6780 ~ 0.8576, sensitivity = 47.27%, specificity = 100%, cutoff value = 1.4727) and 0.8753 (95% CI = 0.8261 ~ 0.9244, sensitivity = 69.23%, specificity = 100%, cutoff value = 1.6923), respectively. In vitro high‐glucose treatment showed that the SRGN expressions were dramatically increased. The loss of SRGN could partially counteract the inhibition of HREC proliferation caused by high‐glucose stimulation. Meanwhile, SRGN knockdown could reverse the promotion of HREC apoptosis induced by high glucose as well.

**Conclusions:**

Consequently, our study implied that SRGN might serve as a promising biomarker with high specificity and sensitivity in the DR diagnosis and progression.

## Graphical abstract

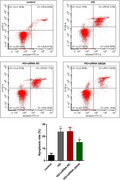

## 1 INTRODUCTION

Diabetic retinopathy (DR), a microvascular complication caused by chronic diabetes mellitus (DM), is a significant threat to vision in adults, which may even lead to blindness and retinal detachment.^1^ The clinical symptoms of DR include fibrosis, capillary obstruction, neovascularization, and increased vascular permeability.^2^ According to the lesion's severity, DR could be classified as proliferative DR and non‐proliferative DR.^3^ Epidemiological studies showed that there were more than 285 million DM patients worldwide.^4^ It was estimated that about 92.4 million adults in China have DM; 43% of them developed DR.^4^ DR was broadly classified into two types: non‐proliferative diabetic retinopathy (NPDR) and proliferative diabetic retinopathy (PDR).^5^ The NPDR stage was mainly characterized by damage to the microvasculature such as massive apoptosis of retinal pericytes and endothelial cells, causing local ischemia, ischemia in the retina, increased vascular permeability, capillary dilatation, and microaneurysm formation.^6^ When neovascularization broke through the inner boundary membrane of the retina to form pathological neovascularization, it was called the PDR stage, which was also the main period of blindness from DR.^7^

Meanwhile, almost all type 1 diabetes cases and more than 60% of type 2 diabetes (T2DM) cases within two decades of onset had DR complications.^4^, ^8^ More importantly, only one‐third of T2DM patients were diagnosed with DR symptoms at admission.^8^ With the lapse of time, when fragile new blood vessels formed on the surface of the retina, the DR process had already progressed to an advanced stage, causing severe visual impairment and even blindness.^9^ Therefore, given the constant increase in DR caused by T2DM, clinical strategies should be developed to identify and treat this complication as early as possible.

To date, the examination of glycosylated hemoglobin (HbA1c), fructosamine, and glycosylated albumin index has been widely used in the clinical diagnosis of the DR level.^10^ However, the specificity, sensitivity, and accuracy of these indicators are limited, and the expenses related to diagnosis constitute a significant challenge.^11^ Therefore, finding novel and accurate factors related to the DR pathogenesis and contributing to clinical diagnosis and treatment is a severe challenge.

Recently, several genes have been identified as having a potential diagnostic and prognostic value in DM and related complications such as diabetic nephropathy and diabetic cardiomyopathy.^12^, ^13^, ^14^ In the realm of DR, various genes were also found to be potential candidates for DR diagnosis and progression. For instance, Li et al found that angiogenesis growth factors VEGFC, ANGPT1, ANGPT2, and EFNB2 were upregulated in DR patients, presenting their potentials as biomarkers for DR diagnosis and treatment.^15^ Another report demonstrated that netrin‐1 and netrin‐4 were overexpressed in the DR group, functioning as promising therapeutic agents for DR.^16^ Serglycin (SRGN) was a hematopoietic granulosa protein‐polysaccharide composed of a relatively small (*17 kDa) core protein.^17^ Studies showed that SRGN was mainly expressed in normal hematopoietic cells, endothelial cells, uterine decidua, and embryonic stem cells.^18^, ^19^, ^20^, ^21^, ^22^

Meanwhile, SRGN was also involved in forming mast cell secretory granules and the localization of granular proteins and mediating the storage and secretion of various proteases, chemokines, and cytokines.^23^, ^24^ Furthermore, novel evidence suggested that SRGN may address an essential role in cancer development. For instance, elevated SRGN expression levels were deemed an unfavorable indicator in primary nasopharyngeal carcinoma.^25^ Furthermore, abnormal expression of SRGN was associated with tumor progression and metastasis in breast cancer and non‐small cell lung cancer.^26^, ^27^ According to a previous study, SRGN was abundantly represented in DN patients' fibrovascular membranes, implying its potential value as a parameter for predicting DR occurrence and development.^28^ However, the clinical and cellular mechanisms of SRGN remain not fully understood. Consequently, the present study aimed to explore whether SRGN might be a candidate for DR's diagnosis and treatment.

## 2 MATERIALS AND METHODS

### 2.1 Sample collection

Our study participants were recruited between December 2016 and March 2018 at Ningbo Eye Hospital, including 130 DR patients (64 PDR patients and 66 NPDR patients), 55 type 2 diabetes mellitus (T2DM) patients, and 46 healthy controls. The inclusion criteria were as follows: diagnosed by color Doppler ultrasonography without drug therapy, and PD caused by T2DM. NPDR and PDR were diagnosed using fundus photography and fluorescein angiography according to the international classification criteria for DR. The exclusion criteria were as follows: severe cardiovascular diseases, hepatic insufficiency, renal injury, infectious diseases, systematic diseases, and tumors.

All plasma samples were collected from peripheral venous blood after 8 h of fasting. After collection, plasmas were centrifugated at 1600 g for 15 min at 4°C and stored at −80°C until further analysis. Written informed consent was obtained from each patient and participant. The ethics committee approved the present study following the Declaration of Helsinki.

### 2.2 qPCR analysis

The expressions of SRGN in plasma samples and cells were measured by qPCR analysis. In brief, total RNA was extracted using TRIzol reagent (Invitrogen; Thermo Fisher Scientific, Inc, Waltham, MA, USA) following the manufacturer's protocol. Then, the RNA was reverse‐transcribed into cDNA using a Reverse Transcription Kit (Life Technologies; Thermo Fisher Scientific Inc, Waltham, MA, USA) and amplified with PCR instrument by SYBR Premix Ex Taq II Reagent (Thermo Fisher Scientific Inc, Waltham, MA, USA) on an ABI PCR System (Applied Biosystems, Bedford, MA) following the manufacturer's instructions. Afterward, the PCR has proceeded at 95℃/30s (40 cycles/92°C for 5 s, 62°C for 20 s, and 72°C for 30 s). β‐actin functioned as an internal control, and the expression of SRGN was normalized to β‐actin using the 2^−ΔΔCq^ method. All primers were designed and synthesized by Gene Pharma (Shanghai, China). The primers were as follows: SRGN forward, 5′‐CAGGTATTCAAGGTCCCATTTCA‐3′ and reverse, 5′‐GGACTACTCTGGATCAGGGTT‐3′; β‐actin forward, 5′‐AACCCTAAGGCCAACAGTGAAAAG‐3′ and reverse, 5′‐TCATGAGGTAGTCTGTGAGGT‐3′.

### 2.3 Western blot assay

Total proteins were isolated from plasma samples and cells using RIPA reagent (Sigma‐Aldrich, USA), and the protein concentrations were determined by a Bicinchoninic Acid Protein (BCA) Assay Kit (Abcam, Shanghai, China). 20 μg proteins were separated by 10% SDS‐PAGE (Thermo Fisher Scientific Inc, Waltham, MA, USA) and transferred to a polyvinylidene difluoride (PVDF; Sigma‐Aldrich, USA) membrane. The membrane was then blocked by 5% non‐fat milk for 1 h at about 25°C and incubated with primary antibodies: rabbit anti‐SRGN (1:1000, ab156991; Abcam, Shanghai, China) and β‐actin (1:1000, ab8227; Abcam, Shanghai, China) at 4°C overnight. Afterward, the membrane was washed with PBS (0.1% Tween‐20) three times and then incubated with IgG H&L (HRP) secondary antibody (1:1000, ab7090; Abcam, Shanghai, China) at room temperature for 2 h. β‐actin functioned as an internal control. ImageJ version 1.46 software (National Institutes of Health, Bethesda, MD, USA) was applied to quantify the band intensity, and relative SRGN protein expression was normalized to the band intensity of β‐actin.

### 2.4 Cell culture and transfection

Human retinal endothelial cells (HRECs) were obtained from Cell Bank of the Chinese Academy of Science (Shanghai, China) and cultured in Dulbecco's modified Eagle's medium (DMEM) supplemented with 10% fetal bovine serum (FBS) and 1% penicillin‐streptomycin at 37℃ in a humidified atmosphere with 5% CO_2_. Then, HRECs were treated with high glucose (30 mmol/L), cultured in standard cell culture conditions until use.

Human retinal endothelial cells were transfected with 40 nM siRNA‐NC or siRNA‐SRGN designed by Gene Pharma (Shanghai, China) using the Lipofectamine 3000 Reagent (Invitrogen; Thermo Fisher Scientific, Inc, Waltham, MA, USA) according to the manufacturer's protocol. The transfected cells were cultured at 37℃, 5% CO_2_ until further analysis.

### 2.5 CCK‐8 cell proliferation assay

After transfection, cells were seeded at a 96‐well plate with a density of 3 × 10^3^ cells/well. When cells approached 70 ~ 80% confluence, transfected cells were treated with high glucose as described above and cultured for another 24 h at room temperature. Subsequently, 10 μl CCK‐8 reagent (Takara, Beijing China) was added to each well for another 2‐h incubation at 37°C. The absorbance of each group was measured at 450 nm using a spectrophotometric plate reader.

### 2.6 Flow cytometric and cell apoptosis assay

After transfection, cells were seeded at a 6‐well plate with a density of 5 × 10^4^ cells/well. When cells approached 70 ~ 80% confluence, transfected cells were treated with high glucose as described above and cultured for another 48 h at room temperature. Subsequently, cells were digested with 0.25% trypsin and stained with FITC Annexin V Apoptosis Detection Kit (BD Biosciences, San Diego, CA, USA) according to the instructions on a FACS Vantage Flow Cytometer System (BD Biosciences, San Diego, CA, USA). The apoptosis data were analyzed by CellQuest software (BD Biosciences, San Diego, CA, USA) to quantify the cell apoptosis ratio.

### 2.7 Statistical analysis

SPSS 21.0 (SPSS Inc, Shanghai, China) and GraphPad Prism 6.0 software were used to analyze the data. All the data obtained in the present study were conducted at least in triplicate. Differences among three groups or more groups were compared and analyzed using one‐way ANOVA followed by Tukey's test. ROC curve analysis was conducted to verify the diagnostic value of SRGN for discriminating DR cases from healthy controls. Differences were considered statistically significant if the *p*‐value was <0.05.

## 3 RESULTS

### 3.1 Clinical features

The clinical parameters of the DR patients, T2DM patients, and healthy controls are presented in Table 1. As shown in Table 1, there were significant differences in fasting blood glucose (FBG), HbA1c, and SRGN levels among the three groups (*p* < 0.001). However, there were no differences in age, gender, body mass index (BMI), diastolic blood pressure (DBP), systolic blood pressure (SBP), total cholesterol (TC), triglyceride (TG), high‐density lipoprotein cholesterol (HLD‐C), low‐density lipoprotein cholesterol (LDL‐C), serum creatinine (Scr), and blood urea nitrogen (BUN) levels.

**TABLE 1:** Clinical features of recruited participants in this study

Features	Healthy (*N* = 46)	T2DM (*N* = 55)	DR (*N* = 130)	*p*‐value
Age (years)	55.8 ± 6.3	56.2 ± 5.5	57.0 ± 5.2	0.3802
Gender				
Male	69	28	24	0.9638
Female	61	27	22	
BMI (kg/㎡)	27.6 ± 2.5	27.3 ± 2.2	26.8 ± 2.6	0.1326
DBP (mmHg)	83.8 ± 7.8	84.9 ± 7.6	85.2 ± 8.1	0.5884
SBP (mmHg)	133.5 ± 11.8	135.8 ± 10.7	134.3 ± 10.9	0.5543
FBG (mmol/L)	4.71 ± 0.78	8.99 ± 1.03	9.07 ± 1.51	**<0.001**
HbA1c (%)	5.09 ± 0.77	7.42 ± 0.92	7.95 ± 1.13	**<0.001**
TC (mmol/L)	4.40 ± 0.96	4.53 ± 0.84	4.51 ± 0.91	0.7320
TG (mmol/L)	1.69 ± 0.64	1.81 ± 0.72	1.92 ± 0.58	0.0918
HDL‐C (mmol/L)	1.27 ± 0.20	1.31 ± 0.17	1.26 ± 0.22	0.3162
LDL‐C (mmol/L)	2.76 ± 0.51	2.89 ± 0.64	2.97 ± 0.61	0.1219
Scr (μmol/L)	56.84 ± 14.02	58.84 ± 12.34	55.01 ± 11.97	0.1558
BUN (mmol/L)	56.84 ± 14.25	57.57 ± 12.97	55.83 ± 10.93	0.6530
SRGN level (fold)	2.08 ± 0.23	1.43 ± 0.12	0.98 ± 0.13	**<0.001**

Abbreviations: BMI, body mass index; BUN, blood urea nitrogen. DBP, diastolic blood pressure; FBG, fasting blood glucose; HDL‐L, high‐density lipoprotein cholesterol; LDL‐C, low‐density lipoprotein cholesterol; SBP, systolic blood pressure; Scr, serum creatinine; TC, total cholesterol; TG, triglyceride.

Bold values indicate *p*<0.001.

### 3.2 Comparison of SRGN levels in plasma samples of NPDR cases, PDR cases, T2DM cases, and healthy controls

**FIGURE 1:**
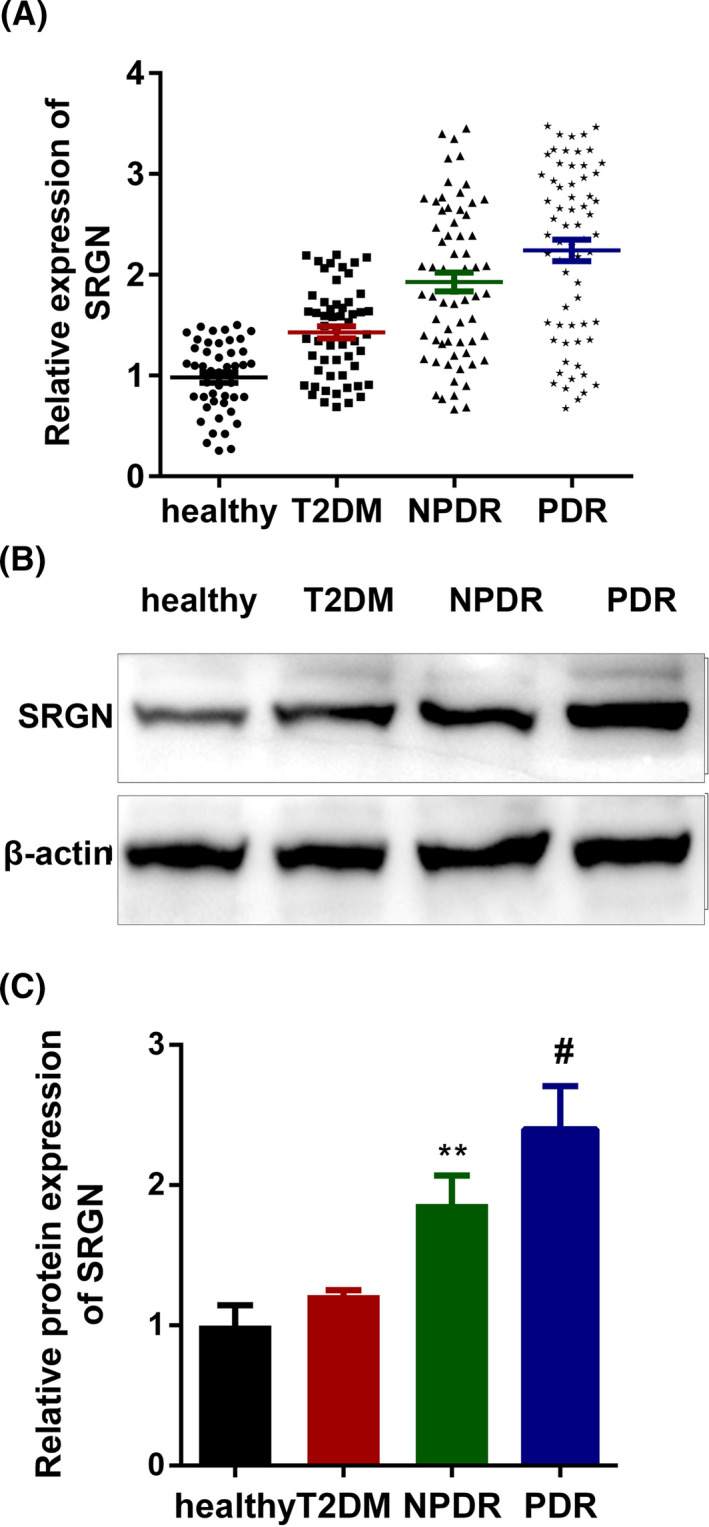
Comparison of SRGN levels among plasma samples from NPDR patients, PDR patients, T2DM patients, and healthy controls. SRGN was significantly increased in DR cases compared with T2DM patients and controls. A, mRNA expression of SRGN measured by qPCR assay. B, Protein bands of SRGN measured by Western blot assay. C, The quantified result of B was presented. ^**^ *p* < 0.01, NPDR vs. T2DM group or healthy group; ^#^ *p* < 0.01, PDR vs. T2DM group or healthy group. NPDR, non‐proliferative diabetic retinopathy; PDR, proliferative diabetic retinopathy; T2DM, type 2 diabetes mellitus; healthy, healthy volunteers

The mRNA and protein expressions of SRGN in plasma samples were measured by qPCR and Western blot assays. As shown in Figure 1, SRGN was dramatically increased in the NPDR group and in the PDR group compared with healthy controls and T2DM patients in both mRNA and protein levels. Furthermore, the level of SRGN was further increased in PDR cases with regard to NPDR group.

### 3.3 The diagnostic potential of SRGN for DR

**FIGURE 2:**
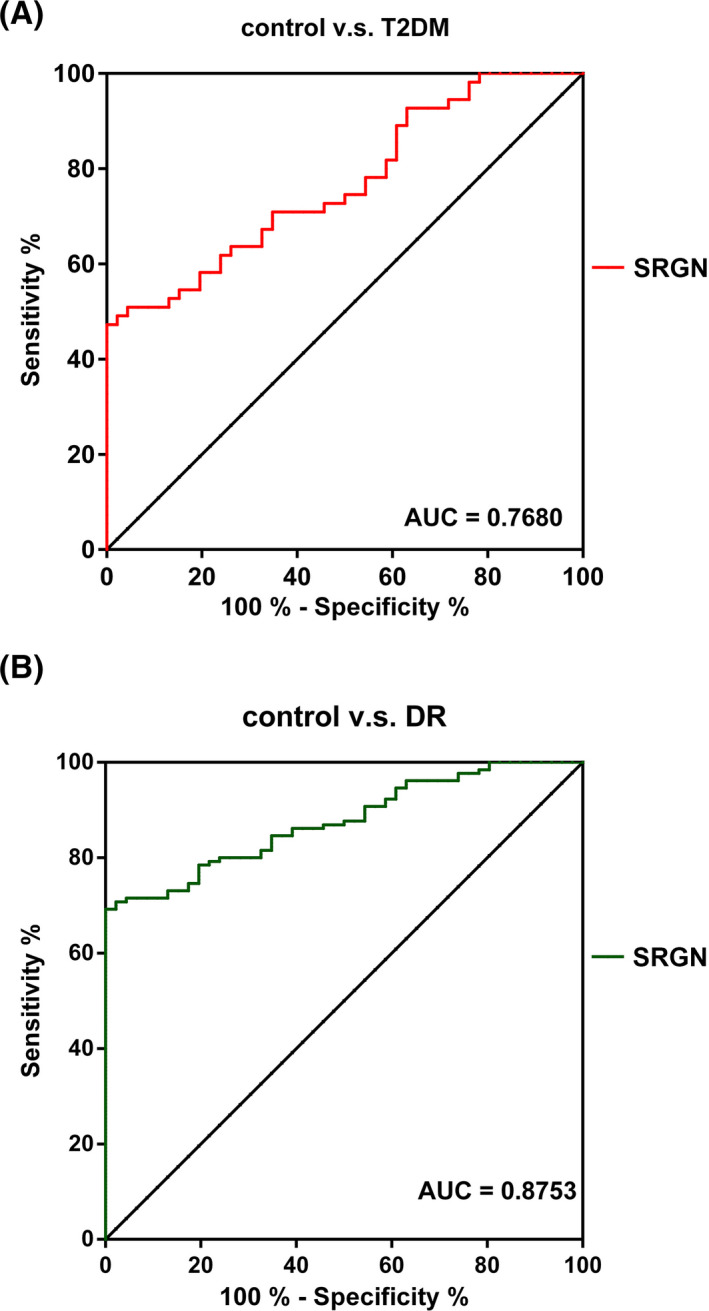
ROC analysis of SRGN for the diagnosis of DR. A, The potential of SRGN for distinguishing DR patients from T2DM patients. AUC = 0.7680, the sensitivity of 47.27%, specificity of 100%, and cutoff value of 1.4727. B, The potential of SRGN for distinguishing DR patients from healthy controls. AUC = 0.8753, the sensitivity of 69.23%, specificity of 100%, and cutoff value of 1.6923. ROC, receiver operating characteristic

According to ROC curve analyses, the diagnostic sensitivity and specificity of SRGN for DR were measured. As shown in Figure 2, the area under the curve (AUC) of SRGN from discriminating DR from T2DM was 0.7680 (95% CI = 0.6780 ~ 0.8576; *p* < 0.01), with the sensitivity of 47.27%, specificity of 100%, and cutoff value of 1.4727; meanwhile, the AUC of SRGN from discriminating DR from healthy controls was 0.8753 (95% CI = 0.8261 ~ 0.9244; *p* < 0.01), with the sensitivity of 69.23%, specificity of 100%, and cutoff value of 1.6923.

### 3.4 Comparison of SRGN levels in high‐glucose‐treated cells

**FIGURE 3:**
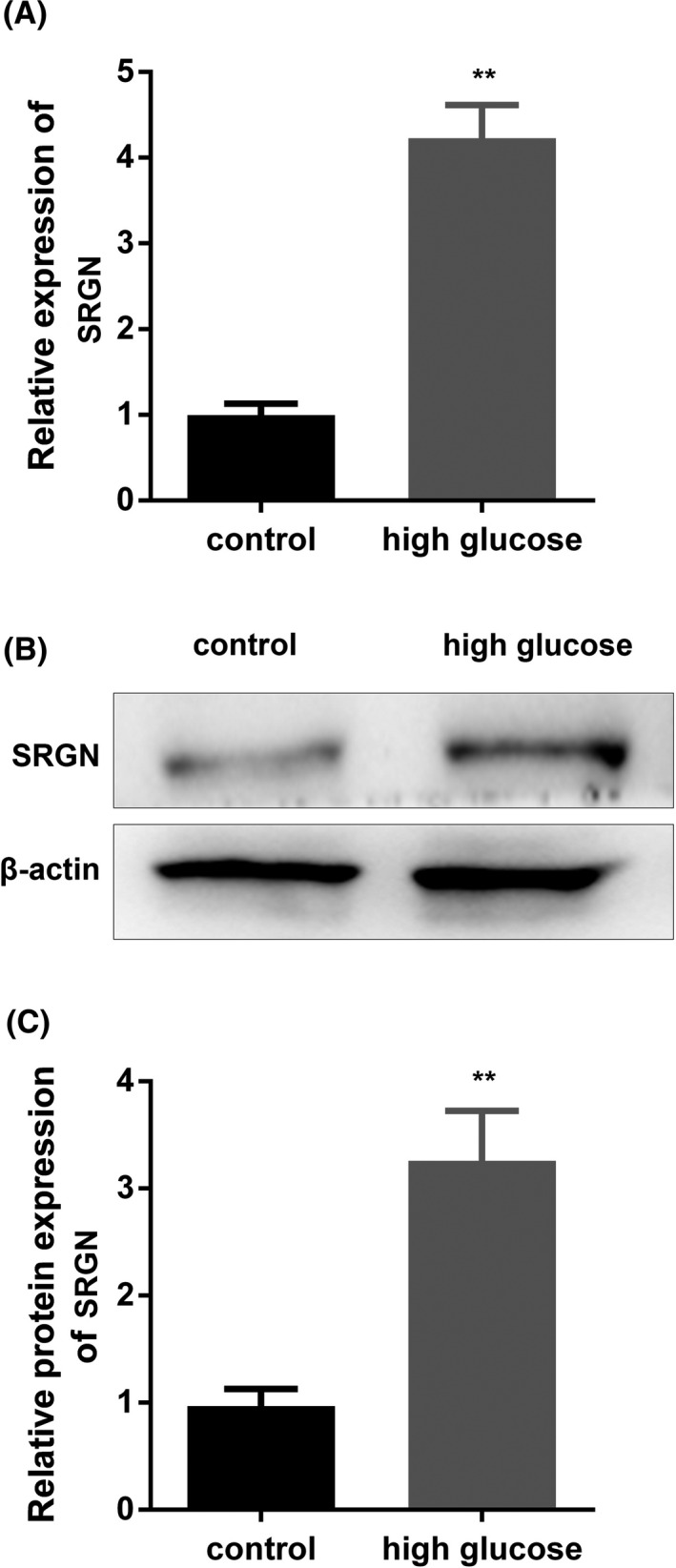
Comparison of SRGN levels in HRECs after treated with glucose. A, mRNA expression of SRGN measured by qPCR assay. B, Protein bands of SRGN measured by Western blot assay. C, The quantified result of B was presented. ^**^ *p* < 0.01, high glucose vs. control group. Control, untreated HRECs with 5.5 mmol/L glucose; high, HRECs treated with 30 mmol/L glucose; HRECs, human retinal endothelial cells

To identify the mechanisms of SRGN at the cellular level, we further constructed a high‐glucose‐treated cell model to address further experiments. After cell culture, we found a significant increase in SRGN expression in high‐glucose‐treated HRECs compared with the control group, which contains average glucose concentration (Figure 3).

**FIGURE 4:**
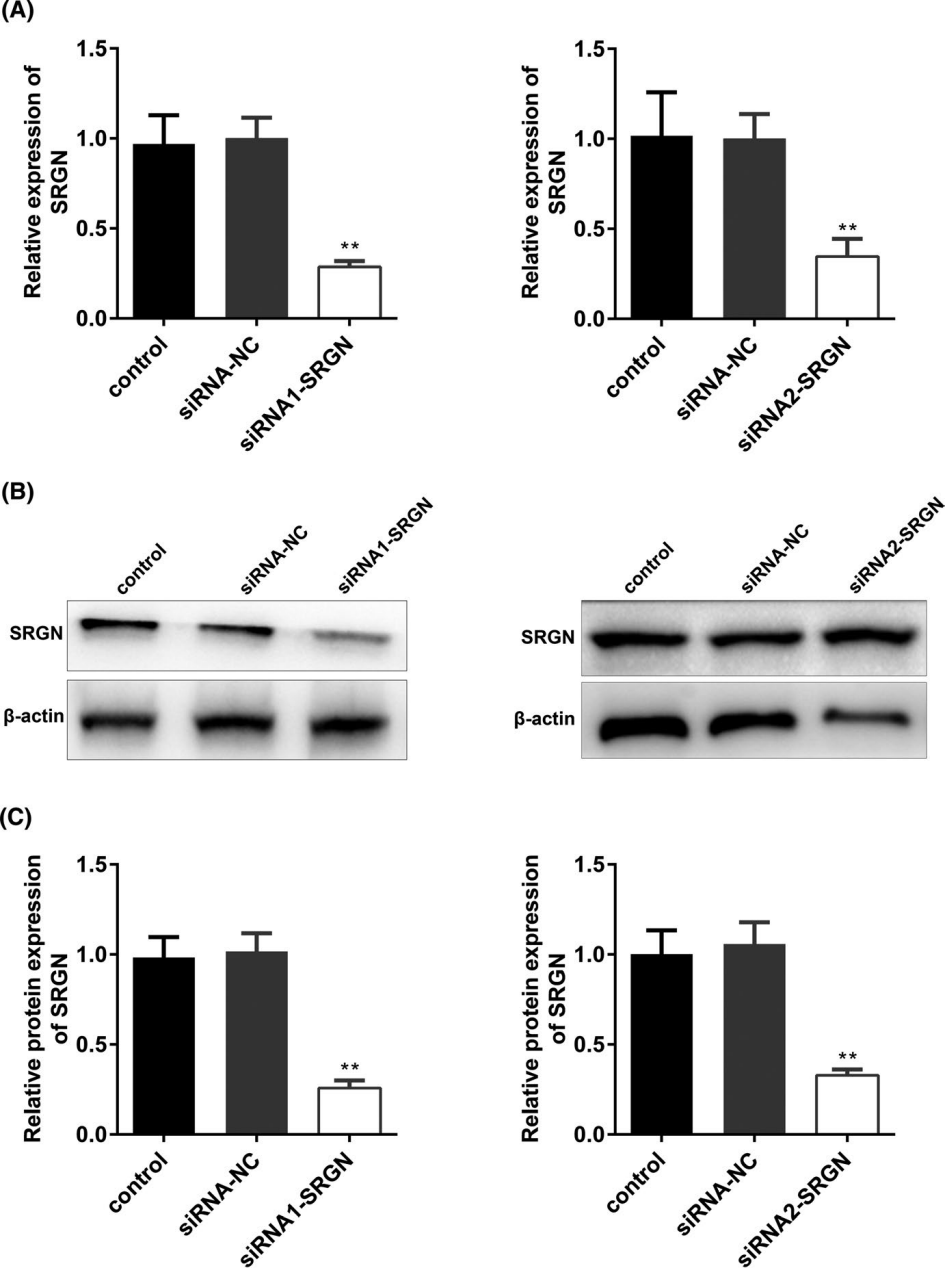
Transfection efficiency of SRGN. A, mRNA expression of SRGN measured by qPCR assay. B, Protein bands of SRGN measured by Western blot assay. C, The quantified result of B was presented. ^**^ *p* < 0.01, siRNA1(2)‐SRGN vs. siRNA‐NC group. Control, untreated HRECs with 5.5 mmol/L glucose

According to the cell transfection data, the expression of SRGN was significantly decreased in the siRNA1‐SRGN and siRNA2‐SRGn groups, suggesting the cell transfection was successful (Figure 4).

### 3.5 Knockdown of SRGN significantly increased proliferation in high‐glucose‐treated HRECs

**FIGURE 5:**
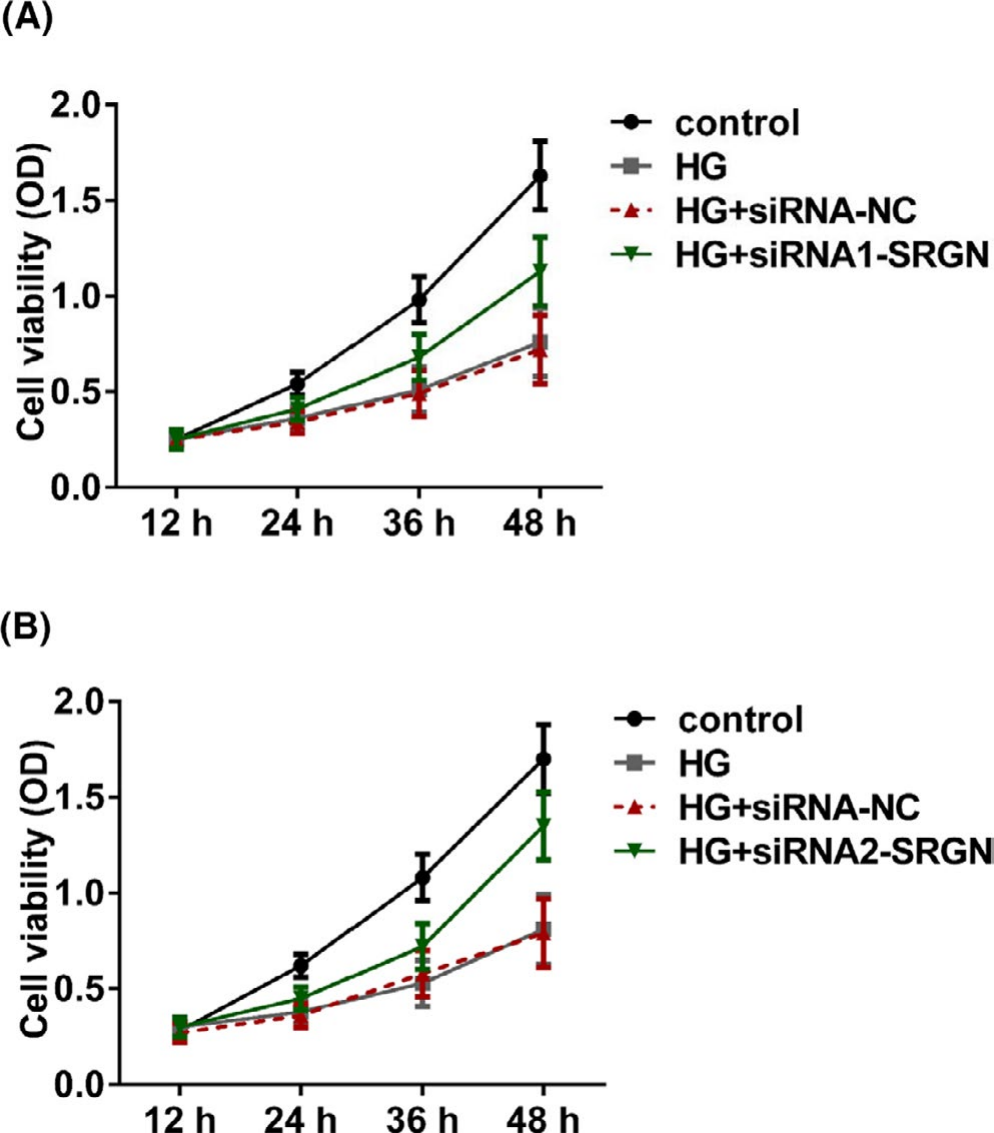
The knockdown of SRGN significantly promoted HREC proliferation after high‐glucose treatment. A, Cell proliferation after treated with high glucose and transfection with siRNA1‐SRGN. B, Cell proliferation after treated with high glucose and transfection with siRNA2‐SRGN. Control, untreated HRECs with 5.5 mmol/L glucose

After transfection and treated with high glucose, cell proliferation was measured by CCK‐8 assay. As shown in Figure 5, after high‐glucose treatment, HREC proliferation was significantly inhibited; however, the inhibition could be partially reversed by transfection with siRNA1‐SRGN or siRNA2‐SRGN.

### 3.6 Knockdown of SRGN significantly decreased apoptosis in high‐glucose‐treated HRECs

**FIGURE 6:**
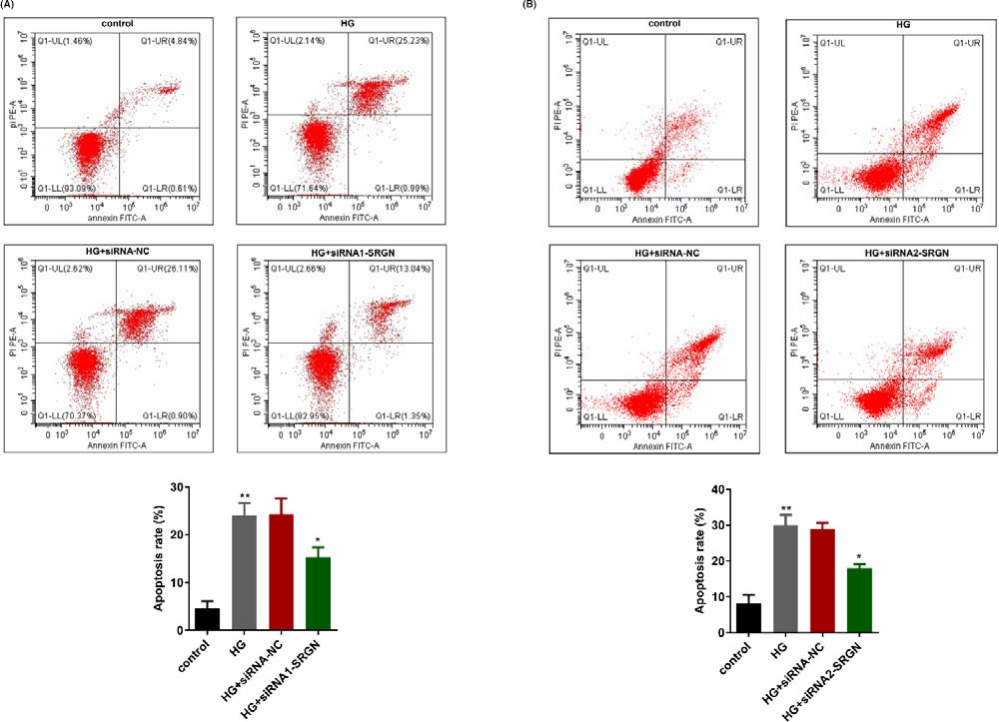
The knockdown of SRGN significantly inhibited HREC apoptosis after high‐glucose treatment. A, The cell apoptosis after treated with high glucose and transfection with siRNA1‐SRGN. B, The cell apoptosis after treated with high glucose and transfection with siRNA2‐SRGN. ^*^ *p* < 0.05, HG + siRNA1(2)‐SRGN vs HG + siRNA‐NC group; ^**^ *p* < 0.01, HG vs control group; control, untreated HRECs with 5.5 mmol/L glucose

After transfection and treated with high glucose, cell apoptosis was measured by flow cytometry analysis. As shown in Figure 6, after high‐glucose treatment, HREC apoptosis was significantly facilitated; however, the promotion could be partially counteracted by transfection with siRNA1‐SRGN or siRNA2‐SRGN.

## 4 DISCUSSION

DR is a multifactorial chronic diabetic eye complication that can eventually lead to microvascular disease, retinal dysfunction, and even blindness.^29^ Reports have shown that DR patients have a higher risk of developing diabetic macrovascular and microvascular complications, which can induce neovascularization, oxidative stress, inflammation, and abnormal cell proliferation and modulation.^30^ It was recently reported that the pathophysiological basis of DR is similar to some chronic changes such as atherosclerosis and obesity, which are considered chronic subclinical inflammation.^31^ DR's pathogenesis is very complex, and studies suggested that DR's occurrence may be related to the imbalance of retinal microenvironment caused by glucose metabolism disorder and endocrine disorder in the human body.^32^

Recent studies proved the potential of SRGN as a biomarker in various diseases. He et al verified that SRGN was significantly elevated in hepatocellular carcinoma patients, representing a crucial marker indicating poor clinical outcomes.^32^ Xu et al demonstrated that the overexpression of SRGN increased colorectal cancer cell migration and invasion and was associated with poor prognosis.^33^ Another study reported that SRGN might be implicated in diabetic tubulointerstitial injury confirmed by multiple‐microarray analysis, which may provide new insights for DN's diagnosis and therapeutics^34^. However, whether SRGN could function as a therapeutic and diagnostic biomarker in DR is not fully illustrated.

This study found that SRGN expression was dramatically increased in plasma samples from DR cases compared with T2DM or healthy participants. Meanwhile, according to the international criteria for DR, SRGN was further elevated in the PDR group compared with the NPDR group. In addition to the SRGN level, biomarkers such as FBG level and HbA1c level showed statistically significant differences among the three groups. This finding was consistent with the previously reported HbA1c level associated with the severity of DR, and the FBG level was also associated with DR's occurrence.^35^ We further used ROC analysis to analyze the diagnostic value of SRGN for DR. As expected, SRGN can differentiate between DR and T2DM patients, and healthy populations with the AUC results from all are higher than 0.8. Therefore, the use of SRGN as a biomarker for DR diagnosis had important clinical significance.

Concerning the in vitro experiments, we established a high‐glucose‐treated HREC model to mimic the intracellular environment of DR. From the results, we found upregulation of SRGN in high‐glucose‐treated HRECs. Then, the knockdown of SRGN could reverse the inhibition of HRECs proliferation caused by high glucose. Meanwhile, blocking of SRGN could also decrease the apoptosis induced by high‐glucose treatment as well.

It is worth noting that this study still has the following limitations. First, we recruited 130 DR patients, 55 T2DM patients, and 46 healthy controls, with relatively small sample size. Simultaneously, participants were all from Ningbo Eye Hospital (Ningbo, China), and the source of samples may be biased. So, in the future, we need to test this in other populations. We completely excluded the effects of unknown confounders, such as clinical testing methods, disease stage, and drug use. Finally, the mechanism by which SRGN affects DR progression at the molecular level needs to be further investigated.

To sum up, in this study, we found that SRGN was significantly upregulated in the serum of DR patients, and after high‐glucose treatment, SRGN promoted the proliferation of human retinal pigment epithelial cells and inhibited the modulation of SRGN. At the same time, SRGN levels were able to screen DR patients from T2DM and healthy populations. Therefore, SRGN may be a potential diagnostic and therapeutic target for DR.

## CONFLICT OF INTEREST

None.

## Data Availability

The datasets used and/or analyzed during the current study are available from the corresponding author on reasonable request.
